# ST‐Segment Elevation Myocardial Infarction With Right Coronary Aneurysm Successfully Treated With a Drug‐Coated Balloon‐Only Strategy

**DOI:** 10.1002/ccr3.70333

**Published:** 2025-03-17

**Authors:** Yu Sugawara

**Affiliations:** ^1^ Cardiovascular Medicine Yamato Kashihara Hospital Nara Japan

**Keywords:** cardiac computed tomography, coronary aneurysm, drug‐coated balloon, ST‐segment elevation myocardial infarction

## Abstract

ST‐segment elevation myocardial infarction (STEMI) with a coronary aneurysm is rare; therefore, its treatment approach remains not well established. This case suggests that drug‐coated balloon angioplasty may be considered for STEMI patients with coronary aneurysms.

Coronary aneurysm incidence ranges from 0.3% to 5.3% [1]. ST‐segment elevation myocardial infarction (STEMI) with a coronary aneurysm as the culprit lesion is rare; hence, no standard therapy or guidelines are established. Drug‐coated balloons (DCBs) are innovative devices used in treating STEMI, allowing stentless procedures with favorable outcomes [2].

We report the case of a 60‐year‐old man with hypercholesterolemia hospitalized for STEMI. His history of Kawasaki disease was unclear. The patient's ultrasensitive troponin T level was 0.030 ng/mL (normal range, 0–0.014 ng/mL). Emergent coronary angiography (CAG) demonstrated mildly ectatic left anterior descending artery (LAD) (Figure 1A, white arrowheads). However, no stenosis or occlusion in the LAD and circumflex artery was observed. Additionally, CAG revealed acute total occlusion of the middle right coronary artery and a circular calcified structure (Figure 1B, red arrows). Thus, percutaneous coronary intervention (PCI) was performed. A 6F Mach1 JR4.0 guiding catheter (Boston Scientific, Marlborough, MA, USA) was placed, and a 0.014‐in. Sion Blue (Asahi Intecc, Akatsuki Cho, Japan) guidewire was introduced. Following recanalization with thrombus aspiration and dilatation with a 2.0 × 15‐mm balloon (Ryurei, Terumo, Aichi, Japan) (Figure 2A), we used intravascular ultrasound (IVUS) (OptiCross, Boston Scientific, Marlborough, MA, USA) (Figure 2B). IVUS revealed a coronary aneurysm with a 180° calcified plaque at the distal segment (Figure 2a) and thrombus at the culprit lesion (Figure 2b, white arrows). The lumen diameter measured 2.45 × 2.87 mm, with a minimum lumen area of 5.47 mm^2^. A 360° vessel wall calcification was found at the proximal segment (Figure 2c), with a lesion length of 15.7 mm (Figure 2B, white arrow). Due to the IVUS findings, PCI was performed with a 3.0 × 20‐mm paclitaxel DCB (SeQuent Please NEO, Nipro, Osaka, Japan) angioplasty following pre‐dilatation with a 3.0 × 13‐mm scoring balloon (Lacrosse aperta NSE, Nipro, Osaka, Japan). The final CAG revealed thrombolysis in myocardial infarction grade 3 flow (Figure 3). Cardiac computed tomography (CT) following PCI showed a 16 × 15‐mm saccular aneurysm (Figure 4A, green lines; Figure 4B, red arrows) with plaques and heavy calcification (Figure 4C, yellow arrows). Aspirin and warfarin were prescribed after discharge to treat atherosclerosis and prevent thromboembolism in the chronic phase.

**FIGURE 1:**
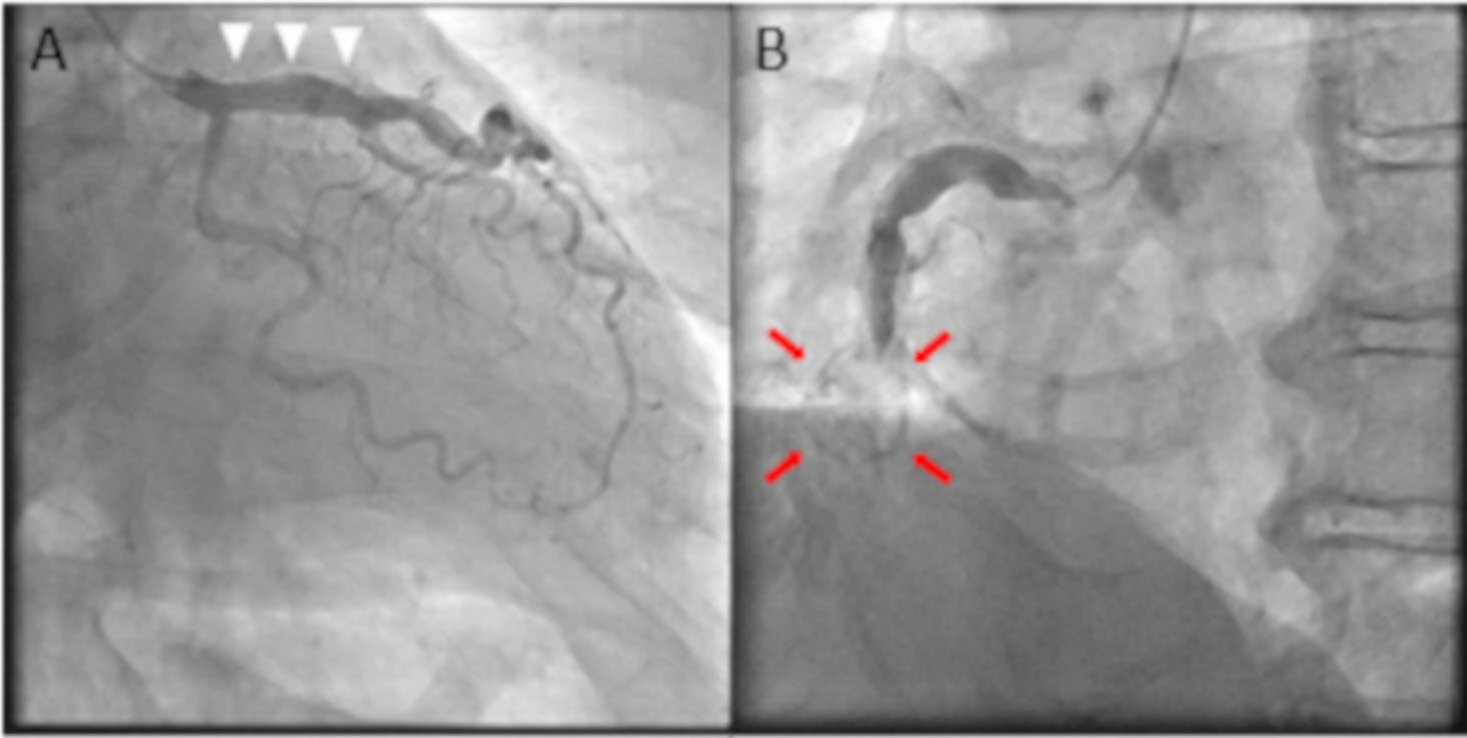
Baseline left and right coronary angiography. (A) The left anterior descending artery (LAD) showed mild dilation (white arrowheads) but no stenosis in both the LAD and the circumflex artery. (B) The right coronary angiography revealed acute total occlusion with the aneurysm's silhouette (red arrows).

**FIGURE 2:**
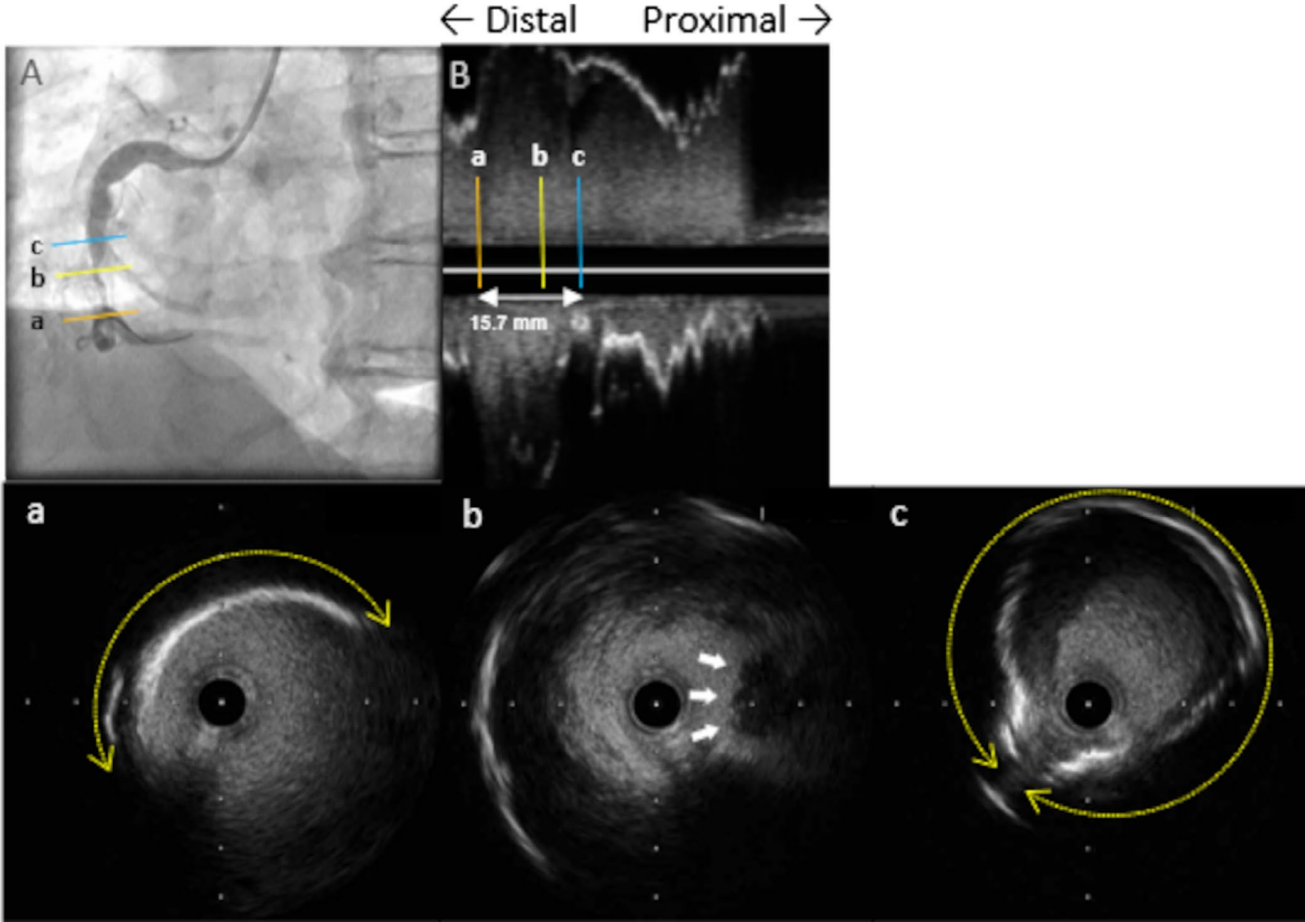
Angiographic and intravascular ultrasound (IVUS) imaging of the right coronary artery after recanalization, showed aneurysm, calcification, and thrombus formation. (A) Right coronary angiography after recanalization. (B) Longitudinal intravascular ultrasound (IVUS) images demonstrated a lesion measuring 15.7 mm in length (white arrow). Each of the a, b, and c lines indicated a cross‐sectional IVUS image. (a) At the distal segment, IVUS showed an aneurysm and 180° calcification (yellow dotted circle). (b) Thrombus formation (white arrows) at the culprit lesion, with a lumen diameter of 2.45 × 2.87 mm and a minimum lumen area of 5.47 mm^2^. (c) At the proximal segment, the vessel exhibited protrusion outward and 360° vessel wall calcification (yellow dotted circle).

**FIGURE 3:**
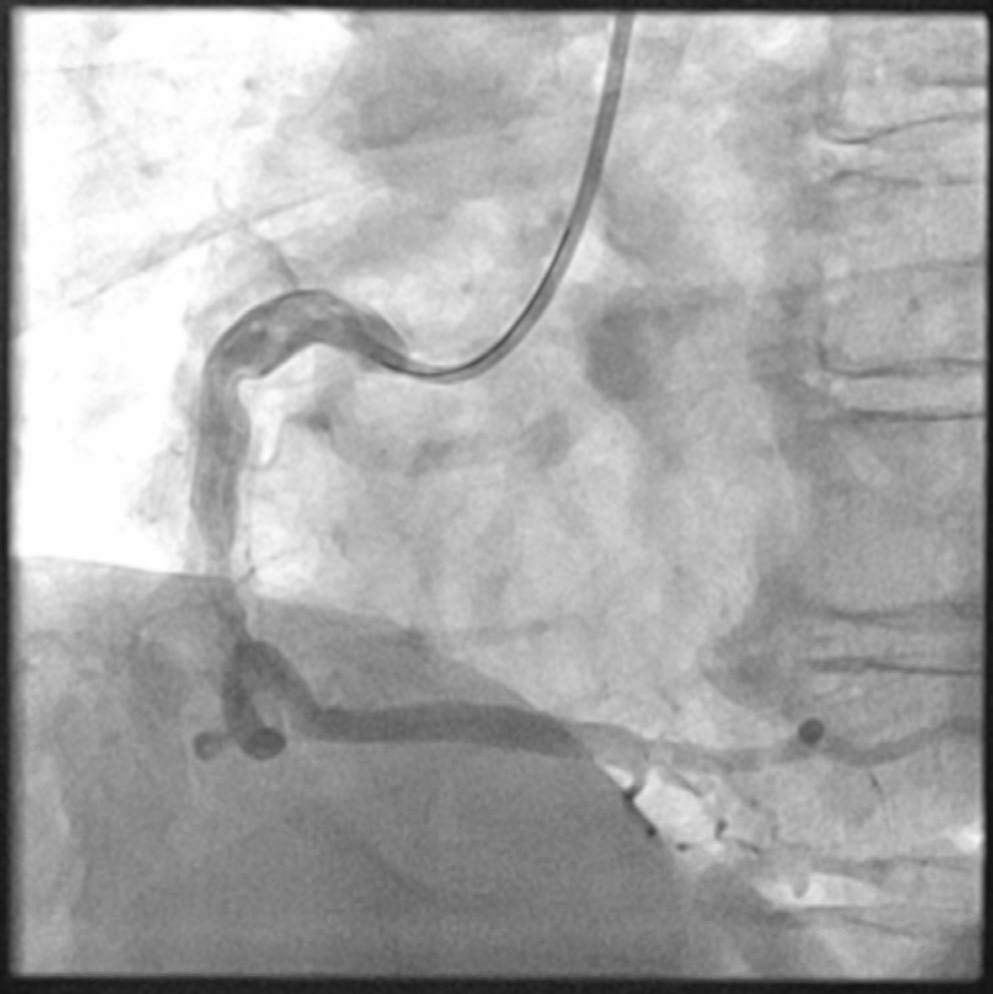
The final angiogram demonstrated a thrombolysis in myocardial infarction grade 3 flow.

**FIGURE 4:**
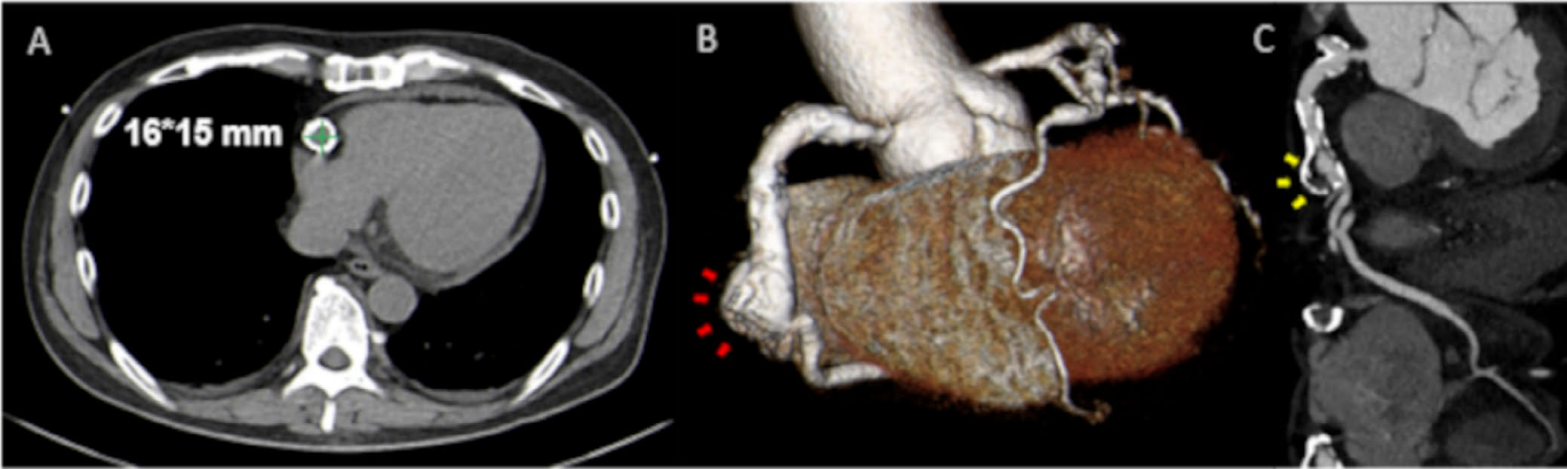
Cardiac computed tomography (CT) after percutaneous coronary intervention. (A) The 16 × 15 mm right coronary aneurysm revealed the entire circumference of calcification (green lines). (B) Cardiac CT demonstrated a saccular aneurysm (red arrows). (C) A saccular aneurysm demonstrated plaque and severe calcification (yellow arrows).

Typically, drug‐eluting stent implantation is recommended in STEMI cases; however, in this instance, a DCB was preferred due to its several advantages. DCBs are non‐inferior to second‐generation drug‐eluting stents in terms of all‐cause mortality and adverse cardiac events in STEMI [2]. Furthermore, DCBs do not trigger in‐stent restenosis or stent thrombus, because they lack the foreign metallic material. The DCB strategy also facilitates cardiac surgery and shortens the duration of dual antiplatelet therapy [3]. Given the potential need for future cardiac surgery (such as aneurysm resection or coronary bypass grafting) and the desire to avoid stent‐related complications, we opted for a DCB‐only strategy.

However, long‐term follow‐up data on STEMI with aneurysm treated using DCBs are lacking, and further cases and studies are necessary to assess the efficacy of this approach.

DCB angioplasty may be considered for STEMI patients with coronary aneurysms.

## Author Contributions

**Yu Sugawara:** conceptualization, investigation, resources, writing – original draft.

## Consent

The author has obtained written informed consent from the patient.

## Conflicts of Interest

The author declares no conflicts of interest.

## Data Availability

All data supporting the findings of this case are available within the paper.
